# Biotechnological Potential of Macroalgae during Seasonal Blooms for Sustainable Production of UV-Absorbing Compounds

**DOI:** 10.3390/md21120633

**Published:** 2023-12-08

**Authors:** Nedeljka Rosic, Carol Thornber

**Affiliations:** 1 Faculty of Health, Southern Cross University, Gold Coast, QLD 4225, Australia; 2 Marine Ecology Research Centre, Southern Cross University, Lismore, NSW 2480, Australia; 3 Department of Natural Resources Science, University of Rhode Island, 120 Flagg Road, Kingston, RI 02881, USA; thornber@uri.edu

**Keywords:** macroalgae, algal bloom, *Sargassum*, *Ulva*, *Gracilaria*, photoprotection, ultraviolet radiation, mycosporine-like amino acids, sunscreens, proteomics

## Abstract

Marine macroalgae (seaweeds) are important primary global producers, with a wide distribution in oceans around the world from polar to tropical regions. Most of these species are exposed to variable environmental conditions, such as abiotic (e.g., light irradiance, temperature variations, nutrient availability, salinity levels) and biotic factors (e.g., grazing and pathogen exposure). As a result, macroalgae developed numerous important strategies to increase their adaptability, including synthesizing secondary metabolites, which have promising biotechnological applications, such as UV-absorbing Mycosporine-Like Amino Acid (MAAs). MAAs are small, water-soluble, UV-absorbing compounds that are commonly found in many marine organisms and are characterized by promising antioxidative, anti-inflammatory and photoprotective properties. However, the widespread use of MAAs by humans is often restricted by their limited bioavailability, limited success in heterologous expression systems, and low quantities recovered from the natural environment. In contrast, bloom-forming macroalgal species from all three major macroalgal clades (Chlorophyta, Phaeophyceae, and Rhodophyta) occasionally form algal blooms, resulting in a rapid increase in algal abundance and high biomass production. This review focuses on the bloom-forming species capable of producing pharmacologically important compounds, including MAAs, and the application of proteomics in facilitating macroalgal use in overcoming current environmental and biotechnological challenges.

## 1. Introduction

Organisms are exposed to diverse levels of ultraviolet radiation (UVR: 280–400 nm) depending on the geographic location. In areas near the equator, the detected UVR levels are extremely high, while UVR levels measured at both poles are very low. Beyond latitude, the seasonal viability in UVR levels also needs to be considered regarding organisms’ capacity to adapt to the periods of high UVR that can be much higher during summer times compared to winter [1]. Additional environmental factors, such as altitude and clouds, could also impact UV levels [2].

Prolonged exposure to UVR may lead to DNA damage, resulting in cellular mutations and long-lasting negative impacts. Humans are especially sensitive to prolonged UVR exposure, leading to more incidences of skin cancers [3,4,5]. Current sunscreens are missing the sufficient sustainable features that are needed for environmental protection. Therefore, natural products (NPs) that could be isolated from species exposed to high levels of UVR are currently very attractive options with clear industrial interest. Potentially promising sources of UV-absorbing NPs come from abundant marine species that are naturally exposed to high UVR, like algae (including macroalgae and microalgae), and can be used to generate sustainable and environmentally friendly sunscreens. 

Marine macroalgae (seaweeds) form the base of many marine and estuarine food webs worldwide. Most members of this polyphyletic grouping belong to one of three main clades: Chlorophyta (green algae), Phaeophyceae (brown algae), and Rhodophyta (red algae). Macroalgae can be found from polar regions to the tropics and range from inhabiting the intertidal zone to nearly 300 m in depth [6,7] in tropical waters (primarily rhodoliths, a group of coralline red algae), although most live in shallower waters of 100 m or less [6]. Macroalgae are all dependent upon specific physical parameters (including light, temperature, salinity, and nutrients) for survival, growth, and reproduction. Macroalgae serve as habitats and/or food sources for a wide variety of marine organisms, modify wave action in coastal areas and serve in blue carbon sequestration pathways [8,9,10,11]. They are also part of a >USD 13 billion dollar global aquaculture industry for human uses, including direct consumption and biomedical and pharmaceutical industries, among others [12,13,14,15]. Macroalgae have evolved to occupy a diverse suite of ecological and environmental niches, with some capable of forming algal blooms. While some species are primarily adapted to cold temperate to polar regions, other groups thrive in tropical locations. Some species have adapted to live in highly stochastic intertidal environments, with diel swings in temperature, salinity and UV exposure, while others occupy much more constant environments in subtidal habitats. Species living in more stochastic environments have evolved with a wide array of defensive compounds and mechanisms. Species in intertidal environments subject to freezing temperatures have evolved to survive the freeze–thaw cycles [16], and most brown macroalgal species have phenolic compounds that protect against a variety of biotic and abiotic stressors [17,18]. Some intertidal species can reduce photosynthetic activity when emersed, which may reduce damage from excess light [19], even though they have increased access to CO_2_ [20]. 

Many macroalgal species living in high-light environments contain mycosporine-like amino acids (MAAs), which are small, temperature- and light-stable, and water-soluble UV-absorbing compounds, with maximal absorption within the range of ~310–360 nm [12,21,22,23]. Strong photoprotective properties and the capacity to absorb light in the UV-A (315–400 nm) and UV-B (280–315 nm) ranges without the generation of harmful free radicals have been confirmed for various MAAs [24,25,26]. Dominant UV-A, which makes ~95% of UV energy reaching the Earth’s surface at moderate levels, has a stimulating role in macroalgal growth and photosynthesis, while UV-B usually has a more harmful impact on marine macroalgae [27,28]. Although UV-A can enhance algal photosynthesis in moderate doses, high levels can reduce quantum yield [29] and inhibit photosynthesis [30], which can be particularly damaging to microscopic stages [31]. Although many species thrive in physiologically stressful habitats, there is still limited understanding and ability to predict the interactive impacts of multiple abiotic stressors on macroalgae [32,33].

**Figure 1:**
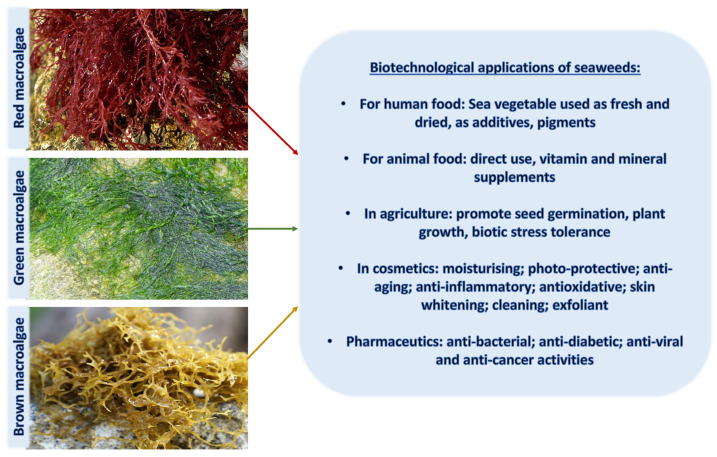
An overview of biotechnological applications of macroalgae in various industries.

**Figure 2:**
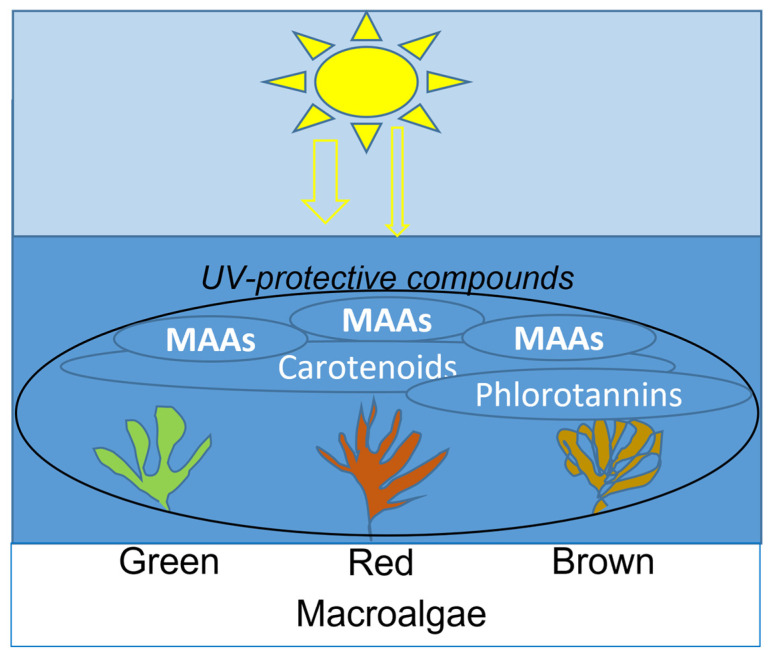
Distribution of main UV-absorbing compounds in macroalgae.

In ancient times, human uses of macroalgae started independently across the globe; Romans used it as food for animals [34], macroalgal remnants have been found in hearths in southern Chile from 12,000 BCE, and medicinal uses of macroalgae were documented nearly 5000 years ago in traditional Chinese herbal medicine [35], as well as many other cultures. Since then, a wide range of applications have been developed, and in modern times, macroalgae have been used in human and animal food, pharmaceutical and other industries, and cosmetics for skin protection due to the presence of components with anti-aging properties, photoprotective, and specifically UV-absorbing capacities (Figure 1 and Figure 2). 

Macroalgae also have an ecologically important role as bioindicators of water quality and have been utilized for bioremediation strategies and the removal of waste products, including heavy metals [36,37]. Pesticides used in local agriculture negatively impact water quality and aquatic ecosystems [38,39], and various macroalgae, such as the red macroalga, *Gracilaria lemaneiformis*, were successfully used for the reduction in insecticide cypermethrin concentration [40], or the brown alga *Saccharina japonica*, for the removal of the herbicide glyphosate in saline waters [41]. 

Human uses of macroalgae have typically focused on either cultivated macroalgae or those harvested from naturally growing populations. Relatively little research has focused on the vast pools of bloom-forming macroalgae, which are sometimes washed ashore (i.e., beach cast or wrack algae), as potential sources of compounds for human usage. Because some bloom-forming species are known to produce UV-absorbing compounds such as MAAs [12], blooms may represent an additional resource for the isolation and purification of these compounds. The aim of this review is to (1) provide an overview of macroalgae forming algal blooms, including the factors triggering these events; (2) determine the biotechnological capacity of selected macroalgal species; (3) evaluate the potential of using these species as a source of UV-absorbing compounds. Furthermore, this review assesses the current application of proteomics for evaluating and utilizing macroalgae capable of forming algal blooms as a sustainable resource for future sunscreens. 

## 2. Macroalgal Blooms

### 2.1. Bloom Overview

Algal blooms are naturally occurring events described as a rapid increase in algal abundance in both microalgae and/or macroalgae, and usually lasting for weeks to months [42,43]. This review is focused specifically on blooms of macroalgae, as bloom-forming species can be found in all three major groups of marine macroalgae [44,45,46]. While many species can occur in both benthic (attached) and pelagic (drift) states, a subset of species typically reach sufficient quantities of drift biomass, characterized as harmful macroalgal blooms. Unlike benthic populations of macroalgae, blooms are characterized by large floating or drifting mats, leading to increased biomass via fragmentation and/or reproduction processes. While some environmental parameters, including light, temperature, and salinity, are well understood to trigger macroalgal booms [47], the exact initiations for a particular species (or group of species) to form a bloom are frequently dependent upon a complex interaction of these abiotic and other biotic factors [48]. 

Harmful macroalgal blooms can have significant adverse environmental and economic impacts on their surroundings. Blooms can impact coral reefs, reduce solar radiation for deeper-dwelling species, hamper gas exchange, and outcompete seagrass [49,50,51,52]. Blooms frequently occur in coastal areas, impeding marine aquaculture, fishing, recreation, and tourism activities. Bloom biomass can foul beaches, ruin fishing gear, and impede fishing, deter tourism, and impede coastal use by a variety of human stakeholders [53,54]. As the location of a particular bloom is impacted by tidal motion, wave energy, and wind direction, their distribution can shift rapidly, making sustained monitoring and/or removal efforts more challenging. In addition, blooms frequently deposit large amounts of biomass on and near coastlines (called beach-cast algae) and subsequently decompose over a period of days to weeks [10,55,56,57]. This decomposition, due to microbial activity, creates hypoxic conditions, harming fish and benthic marine invertebrate communities [58]. In addition, some species release hydrogen sulfide upon decomposition [59] and have a negative impact on carbon sediments [60]. Rather than only serving as a nuisance (or worse) to coastal communities, these beach-cast macroalgae can represent an important source of biomass for human usage. While current efforts typically focus on the removal of beach-cast algae and its subsequent deposition in landfills, the opportunity to use the algae for one (or more) technological applications remains understudied [57]. 

Most bloom-forming species are characterized as being able to survive increased levels of physical stress due to their presence at immediately above or below the waterline. In these environments, they are subjected to increased fluctuations in temperature and salinity (both high and low), as well as higher UV radiation exposure compared to their deeper-dwelling counterparts. Many species of red, green, and brown macroalgae with these characteristics have been documented in blooms, and these blooms are frequently deposited on shorelines, representing a potential for their harvest and utilization. Some of the most prevalent and well-studied blooms include those of the green sea lettuces (*Ulva* spp.), the brown algae *Sargassum* spp. (sometimes referred to as gulfweed), and the red algae *Gracilaria* spp. [45,48,61,62,63]. All three of these taxonomic groups have significant promise for the commercial utilization of bloom biomass [14,64,65,66,67]. Although not covered explicitly in this review, we recognize that many other macroalgal species can form blooms and/or be deposited on beaches in mass quantities, including the genera *Asparagopsis*, *Ecklonia*, *Dasysiphonia* [68,69,70], among many others (see review [57]), which indicates the widespread availability of bloom tissue for potential biotechnological uses. 

### 2.2. Ulva Blooms

The genus *Ulva* contains approximately 100 taxonomically accepted species worldwide, which form thin blades and/or tubes and live predominately in shallow marine and estuarine environments (algaebase.org accessed on 7 June 2023). Blooms of several species of *Ulva* (also known as green tides) occur worldwide in predominantly temperate and subtropical coastal systems. *Ulva* blooms gained worldwide notoriety in 2008 during the Summer Olympics, when their presence threatened the Olympic sailing events [71]. In addition to the Yellow Sea and East China Sea, *Ulva* blooms have been reported near Brittany, France [72], Venice, Italy [73], California and Washington, USA [74], the New England region, USA; [46,75,76], South Africa [77], and the Gulf of California, Mexico [78]. 

*Ulva* blooms are frequently triggered by increases in nutrients on either localized or regional scales [79,80,81], and co-occurring *Ulva* species may react differently to the same environmental triggers [82]. *U. prolifera* blooms in the south Yellow Sea have been linked to the cultivation of nori (the red alga *Pyropia*—previously identified as *Porphyra*) [79,80]. In these blooms, the predominant mechanism of spread originates from attached *U. prolifera* on the floating *Pyropia* aquaculture rafts. These patches then dislodge and drift with prevailing surface currents into the northern Yellow Sea (see summary in Zhang et al. 2017 [80]), and their growth is enhanced by a combination of inorganic and organic nitrogen sources [83]. In Narragansett Bay, Rhode Island USA, *Ulva* blooms frequently contain both *U. compressa* and *U. lacinulata* (as *U. rigida*), and their growth rates are positively impacted by increases in dissolved inorganic nitrogen due, in part, to outputs from sewage treatment plants and changing rainfall patterns [82,84]. Although *U. lacinulata* and *U. compressa* co-occur in blooms, they vary in their growth rates, thermal tolerances, production of allelopathic chemicals, and susceptibility to herbivory [75,85].

*Ulva* can reproduce both sexually and vegetatively through fragmentation, with few top-down controls on its spread [75,86]. Most of the environments where *Ulva* blooms are found are in the intertidal/shallow subtidal, which are high-light (and coupled UV-A and UV-B) conditions [87]. In many ecosystems, *Ulva* bloom biomass is deposited on shorelines due to changes in wind, water currents, and/or tidal patterns [48,80], thereby exacerbating its impacts on coastal communities. 

*Ulva*’s ability to withstand high irradiance has been well documented [87,88], although DNA and photosystem II damage can result [89]. *Ulva* has physiological protective mechanisms that limit the accrual of DNA damage due to UV-B [90], and *Ulva* can undergo photoinhibition during periods of increased UV-B [91]. In addition, some *Ulva* generates higher concentrations of UV-B absorbing pigments than other genera [92]. However, they lack high levels of UV-absorbing compounds like mycosporine-like amino acids and phlorotannins that are found in red and brown macroalgae [93,94]. 

### 2.3. Sargassum Blooms

The brown algal genus *Sargassum* contains many species found in temperate and tropical systems across the globe. However, large floating mats of pelagic *Sargassum* are typically found in the North Atlantic Ocean. There are two species that are most abundant in this region: *Sargassum fluitans* and *S. natans* [66]. The aptly named Sargasso Sea has been reported from the 15th century onwards [45]; it occupies a wide swath of ocean in the North Atlantic sub-tropical gyre. Although the *Sargassum* in the Sargasso Sea is not typically considered to be a bloom due to its longevity, it is characterized by the same qualities of having drifting, large quantities of biomass similar to those found in macroalgal blooms. The *Sargassum* species in the Sargasso Sea are well recognized for their importance as a habitat for numerous fish and invertebrate species as well as food sources [95]. As a genus, *Sargassum* can reproduce sexually or asexually; pelagic *S. fluitans* and *S. natans* reproduce via the latter mechanism, with vegetative growth and division [66], which enhances their potential rate of biomass increase.

In contrast to the long-documented Sargasso Sea, it is only within the past fifteen years that the presence of a ‘Great Atlantic *Sargassum* Belt’ has been identified; this *Sargassum* belt originates in the equatorial Atlantic, not the Sargasso Sea [96]. The annual mega bloom (‘golden tide’) extends from West Africa to the Gulf of Mexico and contains over 20 million tons of *Sargassum* [45,97]; when it reaches coastlines in the Caribbean and Florida (USA) and is deposited on beaches, it can wreak havoc on the environment and economies of local communities [98,99]. Nutrient enrichment is most likely the cause of these blooms, including the introduction of nutrients from increased flooding in the Amazon basin as well as periodic upwelling along the western coast of Africa; as such, golden tides can be viewed as indicators of large-scale eutrophication [100,101]. Another species, *Sargassum horneri*, has been reported to form blooms in the Yellow Sea, indicating that the spread of blooms by this cosmopolitan genus is occurring [61]. 

By living at or close to the ocean’s surface, *Sargassum* is subject to high levels of UV radiation. Many brown algae contain protective antioxidants, including phenolics, carotenoids, and/or isoprenoids [102] to combat the impacts of UV stressors. As a genus, *Sargassum* is no exception to this pattern [103,104]. *S. filipendula*, like other *Sargassum* species, has high antioxidant activity [102]. Species can also undergo structural changes due to UV exposure; *S. cymosum* increases the abundance of phenolic compounds and thickens its cell walls in response to UVR exposure [105]. However, assessments of the physiological properties of pelagic *Sargassum* can be challenging due to logistical constraints, as pelagic *Sargassum* does not grow well in traditional culturing conditions [106]. A recent study of *S. horneri* in the Yellow Sea [107] found that its photosynthetic activity is decreased by exposure to UVR, with higher tissue concentrations of malondialdehyde (MDA) in specimens exposed to UVR. At the same time, the production of carotenoids and UV-absorbing compounds was increased, indicating photoprotective mechanisms that allow *Sargassum* existence in high-light, high-UV conditions at the ocean’s surface. Similarly, pelagic *S. natans* and *S. fluitans* can increase carotenoid production as a result of increased light exposure [108]. In addition, pelagic *S. natans* and *S. fluitans* can release large amounts of dissolved inorganic carbon (DOC), with a high concentration of phlorotannins, a class of polyphenolics [109]. 

### 2.4. Gracilaria Blooms

The red algal genus *Gracilaria* is typically found in intertidal and shallow subtidal estuarine and rocky habitats in tropical and temperate zones and is well known for its bloom-forming capabilities. This highly branched genus can fragment easily and persist while floating in nearshore habitats, increasing its potential for forming large-scale blooms in coastal systems. Two species, *Gracilaria tikvahiae* and *G. vermiculophylla*, have frequently been documented in blooms in the Atlantic Ocean, ranging from Florida to Maine USA [84,110,111], Portugal [112], northern Europe [113] and the Gulf of California, Mexico [63], among other locations. These *Gracilaria* blooms are frequently deposited on shorelines in large amounts [48,114]. Although most studies of *Gracilaria* blooms have focused on these two species, other species, such as *G. tenuistipitata* in Shenzhen Bay, China, have also been documented as forming blooms [115]. Like their bloom-forming counterparts in the green and brown algae, these species live at or near the ocean’s surface and are thus subjected to high light and UV radiation levels. Some species of *Gracilaria* are also cultivated extensively, primarily for agar production or direct human consumption [116]. 

## 3. Macroalgae as a Source of UV-Absorbing Compounds

The biotechnological potential of macroalgae includes the range of molecules from polysaccharides, lipids, proteins, pigments and phenolic composites to various halogenated derivatives [117]. Macroalgae, like many other marine organisms, have been exposed to severe variations in environmental conditions that forced them to adjust, adapt and survive under various external pressures [118]. Macroalgae, like other sessile organisms, experience variable abiotic conditions, including temperature, light irradiance, salinity, and water turbidity, impacting their physiological performance [19,32,51,119,120,121,122,123,124,125]. These stressful conditions especially require adaptability to high levels of UVR, specifically ultraviolet A (UVA; 320–400 nm) and ultraviolet B (UVB; 280–315 nm) [34]. Macroalgal adaptability to extremely high light conditions is facilitated via a range of secondary metabolites, such as different photoprotective pigments (i.e., chlorophyll and carotenoids), as well as UV-absorbing compounds, such as mycosporine-like amino acids (MAAs). Compared to terrestrial plants, macroalgal species have the advantageous capacity to produce biofuels and chemicals due to the renewable nature of these resources [126]. The presence of MAAs in macroalgae was confirmed in 486 species of red algae, 45 species of green algae, and 41 species of brown algae [127]. However, the variability in MAA content and profiles in macroalgae and other marine species are noted to be strongly influenced by the environmental conditions, symbiosis, nutrient bioavailability (e.g., ammonium availability), as well as seasonal changes, especially variation in irradiance levels [128,129,130,131,132,133]. MAAs biotechnological potential is well recognized due to their pharmacological properties, including antioxidant capacities, the ability to suppress singlet oxygen-induced damage [127,129,134,135], anti-inflammatory and anti-aging properties [136,137,138]. To be able to improve the biotechnological application and use of MAAs in cosmetics and for medical purposes, a number of challenges need to be overcome, including the lack of sufficient research regarding the steps necessary for obtaining purified MAA standards, as well as overcoming strong water solubility issues and the low yields of MAAs coming from natural resources. These challenges are in some parts overpowered by an application of various methods for MAA isolation and characterizations [139] and via a use of heterologous expression systems [127,140,141,142] and/or by stimulation of MAA synthesis via specific nutrient and UVR stimulative conditions [90,128,143,144,145,146]. Recovered MAA quantities isolated from different red algae including bloom-forming *Gracilaria* sp. were found to be significantly impacted by extraction solvent used, with the highest yield (increased up to 32.34%) obtained when 25% of ethanol was used compared to other extraction solvents [139]. In addition, there are still significant challenges in harvesting bloom biomass from beach cast areas as well as the water, rapidly separating species of interest from harvested blooms, purifying algal tissues for the extraction of compounds of interest, and conducting all steps with an economically viable approach [57,147,148].

**Figure 3:**
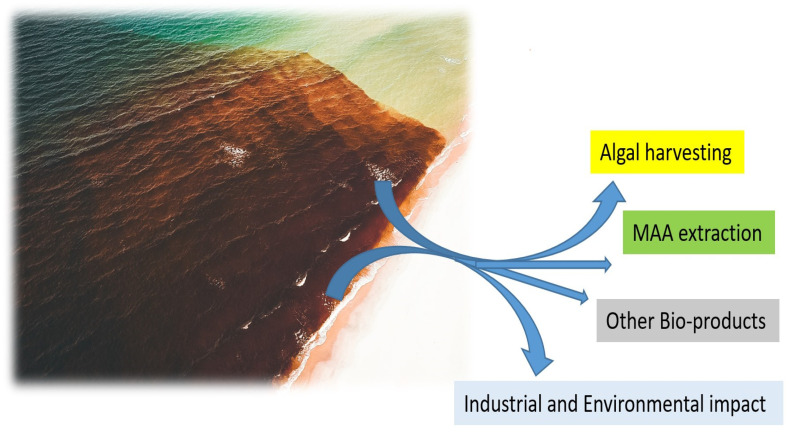
The most economically beneficial source of biomass for massive MAA and other bio-products manufacturing.

Macroalgal species capable of forming algal blooms are especially attractive for biotechnological applications due to the potential economic benefits resulting from their high biomass (Figure 3). Various bioactive compounds can be utilized for different applications ranging from UV protection, food sources, and cosmetics to eco-friendly biopesticides (Table 1). MAA production is confirmed in all three major groups of macroalgae at different profiles and compositions [127]. In green algae, 45 species report detectable levels of MAAs, with the highest quantities identified in species from the class Prasiolales, with MAA content of more than 3.5 mg/g DW, such as reported in *Prasiola crispa* [149], while lower or no detectable MAA quantities are reported in some *Ulva* species [127]. Many macroalgal blooms are dominated by green macroalgae, with the majority of blooms resulting from *Ulva* species [150]. In the case of the bloom-forming genus *Ulva* [151] that leads to overwhelming green tides across the world, mycosporine-glycine and porphyra-334 are confirmed in detectable levels [127]. Biotechnological applications of ulvan beyond MAAs also includes other sulfated polysaccharide with promising anti-cancer, anti-viral, antioxidant and other pharmacological activities [152]. 

Increasing levels of golden algal blooms are reported, especially for *Sargassum* blooms [150]. Brown algae (the class Phaeophyceae) contain the pigments chlorophyll and carotenoids, including fucoxanthin, which is important for photo and antioxidant protection, as well as polyphenolic compounds phlorotannins and complex carbohydrates laminarin and mannitol [153,154]. MAAs are reported in over 40 species coming from brown algae, including some *Saragasumm* species, such as *Sargassum oligocystum* and *S. fluitans* [127]. The invasive species from the genus *Sargassum* (F. Sargassaceae) contain bioactive compounds demonstrating anti-bacterial, anti-inflammatory, antioxidant, anti-tumor, anti-viral, and other pharmacologically promising activities [155], while UVB absorption is confirmed in ethanol extract demonstrating [156] and UVA photoprotective properties [157]. *Sargassum* species are well distributed in tropical and subtropical climate regions and have been used as a source of food for a long time due to their nutrient values (a rich source of vitamins, proteins, and minerals). In addition, pharmacological bioactivities recognized in *Sargassum* species include antioxidant, anti-fouling, anti-microbial, and anti-tumor activities [14]. For example, UV protective, anti-microbial and anti-inflammatory activities are demonstrated in *Sargassum cristaefolium* with confirmed presence of MAA palythene [157,158]. Multiple other MAAs are confirmed in another *Saragassum* species (Table 2). 

Red algae can produce promising bioactive compounds and pigments [14]. Therefore, red macroalgae capable of forming algal blooms are especially attractive due to the high growth and biomass available [117]. On the other hand, the species from the order Bonnemaisoniales, *Asparagopsis armata*, recognized as one of the most aggressive invasive macroalgal species and able to form blooms [159], has very promising biotechnology potential containing various secondary metabolites, including MAAs [117]. *Asparagopsis armata* is becoming very abundant in some countries, presenting a huge negative environmental and economic impact on local communities [160,161]. Although MAA levels are the highest in species coming from the genera *Porphyra* and *Bangia*, the species from the genus *Asparagopsis* are also prosperous with MAA [162], with MAA concentration and profile being directly influenced by nitrogen status [163]. Strong capacity for synthesis of UV-absorbing molecules is reported for *Asparagopsis armata* [163], but not for another invasive bloom-forming species *Dasysiphonia japonica* [44] (order Ceramiales), with species with low content of MAAs (1–2 mg g^−1^ DW). Rhodophyta orders characterized by the highest levels of MAAs of >2 mg g^−1^ DW include Bangiales, Gelidiales and Gracilariales [162]. In 23 red algal species analyzed, the most common MAAs are shinorine, palythine, asterina-330 and porphyra-334 [164]. However, even in red algae, the MAA content may vary from low levels (<1 mg g^−1^ of DW) to higher levels above 2 mg g^−1^ of DW in some species [165]. While the bloom-forming *G. vermiculophylla* contains various MAAs (Table 1 and Table 2), the MAA content and profile can vary seasonally [166]. For cultivated *G. vermiculophylla*, the highest levels of porphyra-334 and shinorine are reported in November–January, while palythine and asterina-330 are highest from April to August. Similarly, when grown in seawater with elevated nutrient concentrations (150 uM of NH_4_^+^ and 15 uM of PO_4_^3−^) and increased UVA and UVB levels, MAA production can increase by 50% in *G. cornea* (old name *Hydropuntia cornea*) [167]. The targeted UV radiation also induces a substantial increase in the MAA levels in macroalga *Gracilaria gracilis* compared to control [143]. Similarly, elevated levels of NO_3_^−^ can increase levels of MAAs in *G. tenuistipitata* [162,168]. Thus, as blooms of *Gracilaria* frequently occur during the summer months when UV exposure is higher and nutrient levels are frequently elevated as well [169], they may represent a high-quality source for obtaining the high biomass levels needed for MAA extraction in the future (Figure 3). 

**Table 1:** Macroalgal species forming algal blooms were reported to accumulate high levels of UV-absorbing compounds.

Macroalgal Species	Biotechnology Use [Ref.]	MAAs
*Green algae* Order: Ulvales *Ulva* spp.	Human and animal nutrients; preservatives; pharmaceuticals; cosmeceuticals	MG, PR [127]
*Brown algae* Order: Fucales *Sargassum cristaefolium* *Sargassum oligocystum*	Photoprotective activity against UVR; Inhibited proinflammatory TNF-*α* and IL-6 expression while increasing IL-10 production in the BALB/*c* mice skin [157,158]	PE dominant MAAs PR, PI, SH 5 [127,170]
*Red algae* Order: Bonnemaisoniales *Asparagopsis armata*	High biofiltration capacity of nutrients; UV photoprotection [163] Exudate cocktail as a biopesticide for eco-friendly weed control [171] Preservatives, cosmeceuticals, biopharmaceuticals [117]	MAAs (accumulated only under a high ammonium-N availability) [163] AS, PR, PE, SH, UN [127]
*Red algae* Order: Gracilariales *Gracilaria vermiculophylla*	Increase in MAAs in freshly released spores increased under UVR 8 h [172]	AS, PE, PR, PI, SH, US, UN [127,166]

Abbreviations: Asterina-330 (AS), Mycosporine-glycine (MG), Palythine (PI), Palythene (PE), Porphyra-334 (PR), Shinorine (SH), Unidentified MAAs (UN), Usujirene (US). Red algae—Rhodophyta; Green algae—Chlorophyta; brown algae—Phaeophyceae.

**Table 2:** Chemical structure of common MAAs found in bloom forming macroalgal species.

UV-Protective Natural Products	Chemical Structure	Key Properties (Ref)	ʎ Max (nm) ε Coefficient (M^−1^ cm^−1^) Molecular Mass (g/mol)
Mycosporine-glycine (C_10_H_15_NO_6_)	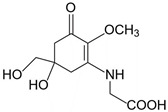	UV-absorbing, antioxidants [134]	310 nm 28,100 M^−1^ cm^−1^ 245 g/mol
Shinorine (C_13_H_20_N_2_O_8_)	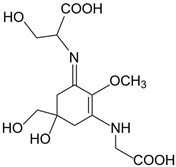	UV-absorbing, antioxidants [135,173]	334 nm 44,668 M^−1^ cm^−1^ 332 g/mol
Usujirene (C_13_H_20_N_2_O_5_)	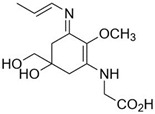	UV-absorbing, antioxidants [174]	357 nm 45,070 M^−1^ cm^−1^ 284 g/mol
Asterina-330 (C_12_H_20_N_2_O_6_)	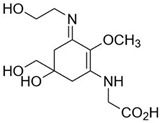	UV-absorbing, antioxidants [175]	330 nm 43,800 M^−1^ cm^−1^ 288 g/mol
Porphyra-334 (C_14_H_22_N_2_O_8_)	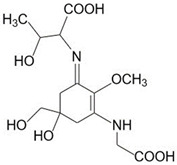	UV-absorbing, antioxidants [135,175]	334 nm 42,300 M^−1^ cm^−1^ 346 g/mol
Palythene (C_13_H_20_N_2_O_5_)	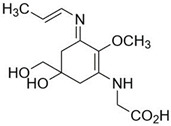	UV-absorbing, antioxidants [176]	360 nm 50,000 M^−1^ cm^−1^ 284 g/mol
Palythine (C_10_H_16_N_2_O_5_)	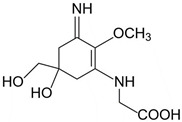	UV-absorbing, antioxidants [175,177]	320 nm 35,500–36,200 M^−1^ cm^−1^ 244 g/mol

## 4. Proteomics for Monitoring Macroalgal Blooms and Discovery of MAA Profiles

The use of transcriptomics and proteomics in bioinformatic pipelines enables faster discovery and functional characterization of novel marine natural products [178]. The exploration of the biosynthesis of MAAs employed genomic mining techniques [142,179], confirming that MAA production occurs via the shikimate pathway [180,181] and the pentose phosphate pathway [179]. Proteomic data analyses demonstrated that UV-induced MAA production mainly occurs via the shikimate pathway and is, therefore, more critical in photoprotection [182]. Four genes making a core of the MAA pathway (e.g., so-called mys cluster genes) were identified to be dehydroquinate synthase (DHQS), O-methyltransferase (O-MT), adenosine triphosphate (ATP) grasp, and nonribosomalpeptide synthetase (NRPS) in the cyanobacterium *Anabaena variabilis* [142]. Some of these genes are duplicated or have additional *mys*-cluster genes in different organisms [129,179,183,184]. Similar observations were reported in other algal groups with mechanisms such as horizontal gene transfer and acquisition of diverse MAA gene clusters playing a driving role in the development of species with high-temperature resilience [185], plus epigenetics mechanisms influencing gene expression patterns [186]. 

Various proteomic techniques can be applied to explore MAA synthesis and monitor macroalgal blooms that could be utilized for the biotechnological application of MAAs. These include high-throughput methods such as top-down proteomic methodologies that separate proteins and then complete individual characterization, such as mass spectrometry (MS)-based proteomics [187]. In bottom-up proteomics (also called ‘shotgun’ proteomics), the proteins first undergo the digestion process, producing a mixture of peptides that are analyzed using MS or LC/MS and compared to existing databases via automated analyses [188]. Most MAA analyses include the purification step, identification and quantification using high-performance liquid chromatography (HPLC) separation and identification based on retention times and UV spectra [189,190,191]. In addition to HPLC chromatography, the confirmatory analyses for improved MAA characterization and quantification also included the implementation of mass spectrometry, including various types of liquid chromatography (LC/MS) methodologies [139,192,193,194,195,196]. The different HPLC and MS techniques improved the discovery and characterization of MAAs, especially when purified MAAs were characterized by nuclear magnetic resonance (NMR) [173,197]. The use of ultrahigh-performance liquid chromatography (UHPLC) was also applied in MAA analyses [198], combined with hyphenated to orbitrap high-resolution tandem mass spectroscopy for feature-based molecular networking characterization and classification of MAAs, which is one of the most recent advancements in proteomics [199]. This approach incorporates the published MAA fragmentation patterns and uses in silico annotation tools that allow for more accurate identification, discovery, and classification of MAAs [199].

Furthermore, predicting algal bloom events could be critical so that the biotechnological industry can utilize these natural events and harvest a large amount of algal biomass as a source of bioproducts. Proteomic studies of differential expression of specific proteins involved in growth processes and stress response can be potentially useful as biomarkers for the prediction of future algal blooms [200]. In China’s coastal waters, distinct genetics patterns were linked to harmful macroalgal blooms (HMBs) involving green and gold tides [201]. Due to the increased negative ecological impact of these HMBs, monitoring these changes and identifying driving regulatory mechanisms is becoming critically important to allow improved scientific forecasting of future algal blooms. Consequently, utilizing other omics datasets was recognized as a promising way to increase the modeling strength for predicting climate-driven algal blooms [202,203]. 

## 5. Conclusions

Macroalgal blooms are spontaneous and frequently brief occurrences usually triggered by anthropogenic factors; they are characterized by a sharp rise in drift macroalgal abundance. Consequently, large amounts of macroalgal biomass can be harvested from these blooms and utilized for biotechnological purposes, although there may be technological challenges associated with the proper harvesting of bloom-forming species. Several macroalgal species that have a strong capacity for both algal blooms and large MAA production were identified in this review. Environmental conditions, including seasonal periods of high UVR exposure and nutrient enrichment, which enhance algal growth, also enhance the accumulation of MAAs, which hold excessive biotechnological potential due to their enhanced photoprotective and other pharmacological properties. Advancing new trends, such as applying a novel molecular networking approach, presents a promising field that combines in silico tools with modern high-throughput chemistry methodologies for analyzing the clusters of MAAs chemistry based on their fragmentation patterns for improved characterization and classifications. Finally, advancements in proteomic techniques can empower our understanding of MAA structural diversity and functional significance for better utilization of natural phenomena of algal blooms and future biotechnological developments (i.e., sunscreens) in an environmentally sustainable manner.

## Data Availability

The original data presented in the study are included in the article; further inquiries can be directed to the corresponding author.
